# Gastroduodenal Ulcerative Colitis in Association with Ulcerative Pancolitis

**DOI:** 10.1155/2021/6684806

**Published:** 2021-02-12

**Authors:** Khin San Aye, Lin Lin Htun, Thet Mar Win, Mya Thida Aye, Drmyothettin Tin, Than Than Aye

**Affiliations:** ^1^Department of Gastroenterology, University of Medicine 2, Yangon, Myanmar; ^2^Department of Pathology, Thingangyun General Hospital, Yangon, Myanmar; ^3^Yangon General Hospital, Yangon, Myanmar

## Abstract

Ulcerative colitis (UC) is a chronic inflammatory bowel disease, traditionally regarded as being limited to the colorectum. Although several gastroduodenal lesions have been reported in cases of UC, in general, duodenal lesions in UC are believed to be uncommon and gastric lesions in UC are a rare presentation. In this report, we presented a 66-year-old lady with upper GI presentation with gastroduodenal ulcerative colitis accompanying pancolonic UC.

## 1. Introduction

Ulcerative colitis (UC) is a chronic inflammatory bowel disease (IBD) of the colon that has an unknown etiology. UC is localized to the colon and spares the upper gastrointestinal (GI) tract. This disease usually begins in the rectum and extends proximally for a variable distance [1]. The annual incidence of UC is 10.4 to 12/100,000 people, and the prevalence is 35 to 100/100,000 people in the USA [2]. Prevalence is lower in Asia [3]. In IBD demographics in a Southern Asia study, a total of 10400 patients were found in 15 countries of South Asia, South East Asia, and the Middle East. A total of 248 IBD patients from Myanmar were included in this study [4]. Although UC is known to have various extracolonic manifestations, the upper GI tract is not generally considered a target organ. The concept of gastroduodenal UC (GDUC) was first proposed by Hori et al., but the diagnosis standard is not rigorous enough due to the scanty reports. Extensive colitis and a lower dose of prednisolone administration might be the main risk factors for GDUC [5]. Several case reports describing upper GI involvement in UC have been published. These reports described gastritis or diffuse duodenitis that resembled colonic lesions of UC in both the endoscopic and pathological findings [6–9]. In a study from Japan, only 4.7% (15 out of 322) of patients had ulcerative gastroduodenal lesions (UGDL) accompanied by pancolitis or after proctocolectomy [10]. There are no RCT and observational studies although several case reports were published because of less number of cases all over the world.

The aim of this report is to present a rare occurrence of the typical UC apparently localized not only to the whole colon but also the upper GI tract, stomach, and duodenum.

## 2. Case Presentation

A 66-year-old previously healthy woman, without UC family history, started to complain of diffuse abdominal pain and decreased appetite since 3 months ago. She also complained of loose motions almost containing mucous and, sometimes, blood more than 10 times per day for 3 months of duration. She also noted significant weight loss of about 10 kg. No other comorbid disease was there except hypertension for 20 years which was controlled with antihypertensive treatment. She underwent colonoscopy in her early onset of symptoms. Colonoscopy finding was multiple polyps throughout the colon, and her doctor advised surgery. However, she refused to undergo surgery and went abroad and sought further management. She underwent upper and lower endoscopy again there, and tissue biopsy was performed from both the GI tract and colon. Biopsy results showed chronic inflammation only and were not conclusive of ulcerative colitis. She was diagnosed as ulcerative colitis based on the endoscopic findings. She received mesalazine 1500 mg twice a day and prednisolone 5 mg twice a day since after diagnosed. Symptoms were improved after treatment. She continued taking medicine without a follow-up visit again.

Two months later, she developed similar episodes of abdominal pain and bloody diarrhea despite taking medicines. She looked pale and had generalized edema. Her heart rate was 112/min, and the blood pressure (110/70 mmHg) and respiratory rate (24/min) were normal. The abdomen was soft; mild tenderness was present in the lower abdomen, but there was no guarding.

Blood investigation showed pancytopenia, hemoglobin 9.2 gm /dl, WBC count 2.75 × 10^3^/ul, and platelet 44 × 10^3^/ul, and hypokalaemia, 2.8 mmol/L. The creatinine level was low (10 umol/L) likely due to low muscle bulk and low serum protein although there were no liver diseases. Low blood calcium level (1.6 mmol/L), low serum phosphate level (0.5 mmol/L), normal magnesium level (0.8 mmol/L), and low serum albumin level (2.1 gm/dl) were found. The blood sugar level was normal. High inflammatory makers such as CRP 447 mg/L, ESR 90 mm/hr, and fecal calprotectin level 827 ug/g were found. Procalcitonin was normal. Serologic testing for antineutrophil cytoplasmic antibodies (ANCA) and antisaccharomyces cerevisiae (ASCA) antibodies were negative in this patient. Stool microscopy showed numerous RBC and pus cells. Blood, stool, and urine culture were negative. Due to previous exposure with steroid hormonal therapy, stool test for CMV and stool test for *Clostridium difficile* for toxin A and B were negative.

CXR showed mild cardiomegaly and small bilateral effusion. CT abdomen remarked as diffuse mucosa-wall thickening throughout the stomach and the first part of the duodenum. Enhancement was noted at the contrast study. The imaging report considered MALT lymphoma. Small amount of ascites were noted in the abdomen and pelvis. Small bilateral effusion was also noted.

Upper and lower endoscopy and biopsy were repeated for the reason of recurrent symptoms, inconclusive of previous histological results although treated as UC and to exclude other differential diagnosis, especially MALT lymphoma.

**Figure 1:**
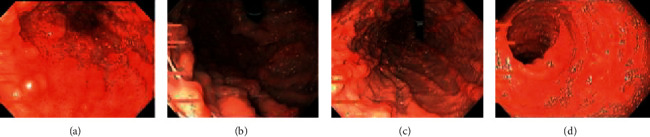
EGD scopy findings; panulcerative gastritis and ulcerative duodenitis. (a) Antrum, (b) fundus, (c) proximal body stomach, and (d) descending part of the duodenum.

Esophagogastroduodenoscopy findings were mucosa granularity, friability with rough and eroded ulcers, multiple inflammatory polyps, and complete obliteration of the vascular pattern diffusely involving the entire GI tract and duodenum. These gastroduodenal findings were similar to that we found in ulcerative lesions of ulcerative colitis in the colon (Figure 1). The rapid urease test for *Helicobacter pylori* infection was negative. Tissue biopsies were taken from the stomach and duodenum separately.

**Figure 2:**
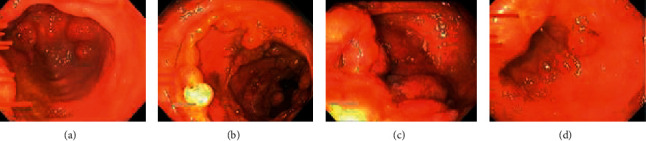
Colonoscopy findings; pancolitis with inflammatory polyps. (a) Rectum, (b) ascending colon, (c) caecum, and (d) terminal ileum.

Colonoscopy findings were mucosa granularity, friability with multiple inflammatory polyps throughout the colon (from the rectum to caecum) with patchy obliteration of the vascular pattern. Inflammations more marked at caecum and ileo-cecal valve. Diffuse mucosal inflammation with obliteration of the vascular pattern shown in terminal ileum is likely backwash ileitis (Figure 2). As the ulcerative colitis endoscopic index of severity (UCEIS) was 9 by 11 and as the Mayo scoring system for assessment of UC activity was 9 by 12 in this patient, it showed severe disease activity since admission.

### 2.1. Histopathologic Report

**Figure 3:**
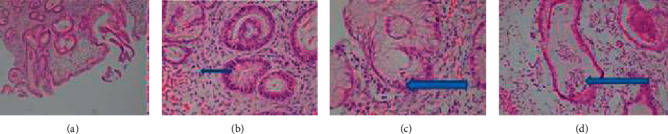
Histologic findings of gastric biopsy; the gastric mucosa shows (a) severe inflammation, ulcerations, and architecture changes, (b) plasmacytosis in the lamina propria, (c) inflammation in the gastric foveolar epithelium, and (d) microabscess formations.

**Figure 4:**
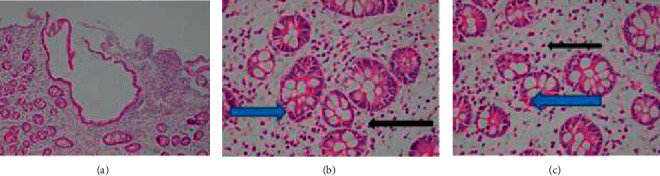
Histologic findings of duodenal biopsy; the duodenum mucosa shows (a) villous atrophy, architecture changes, and edematous lamina propria, (b) plasma cells (black arrow), and (c) cryptitis (blue arrow) in the lamina propria.

Both gastric and duodenum mucosa are severely inflamed with edematous lamina propria and architecture changes. Histological findings are consistent with ulcerative colitis with upper GI involvement. The gastric mucosa shows ulcerations, architecture changes with loss of foveolar pits, basal intense mixed inflammations, and edematous laminopropria. Superficial plasmacytosis are also noted. The glands are cystically dilated and filled with secretions (Figure 3). Microabscesses are appreciated. Granulomas are not seen. The duodenum mucosa shows villous atrophy, architecture changes, edematous lamina propria, and infiltration by lymphocytes and plasma cells (Figure 4). However, neither intraepithelial lymphocytosis nor lesions are appreciated. Epitheloid granulomas are not seen. The histological findings are consistent with inflammatory bowel disease (ulcerativecolitis) with upper GI involvement.

**Figure 5:**
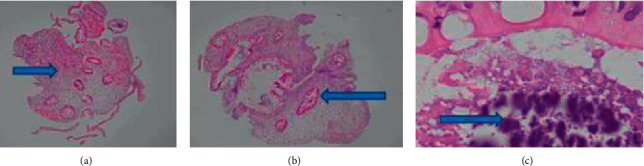
Histologic findings of colon biopsy; all pieces of the colonic polyps show (a) edematous lamina propria, crypt dropout, and mononuclear infiltrates, (b) crypts lined by reactive columnar cells, and (c) bacterial colonies.

The colonic biopsy result was consistent with ulcerative colitis with superimposed bacterial infection. All pieces of colonic polyps showed architecture changes, crypt dropout with edematous stroma and severely inflamed mucosa, and mild diffuse mononuclear infiltrates in the lamina propria. Some crypts are lined by columnar cells with reactive changes. Few bacilli are seen in the crypt lumen. Dysplasia and features of malignancy are not seen. No features of MALT lymphoma were seen (Figure 5).

During hospital stay, she developed many significant events. Right calf muscle pain due to deep vein thrombosis was proved with a vascular Doppler and received anticoagulant, subcutaneous LMW heparin. Fast atrial fibrillation was controlled with amiodarone by the cardiologist. Hypokalaemia, hypomagnesemia, and hypoalbuminemia were replaced according the results. The patient developed refeeding syndrome and was fed according to the nutritionist's advice. The patient could not take oral feeding due to abdominal pain and reduced appetite, which is why parenteral nutrition was given. Pancytopenia was corrected under hematologist guidance. Although there was a clinical association between UC and myelodysplastic syndromes and aplastic anemia, no bone marrow biopsy was performed in this patient. Broad-spectrum antibiotics were prescribed.

The treatment of UGDL is similar to that of UC, so the patient was treated with mesalazine and steroid hormones. Pantoprazole was given concomitant along with intravenous corticosteroid injection. Diarrhea was controlled after 10 days of treatment. Her appetite was improved, and she started to tolerate liquid diet. Oral prednisolone was changed, and it was tried to tail off the drug. Pancytopenia was improved (Hb 9.8 g/dl, WBC 3.8 × 10^3^/ul, and platelet 100 × 10^3^/ul). Serum potassium raised to near normal 3.3 mmol/l. Serum albumin was raised to 3.2 g/dl.

**Figure 6:**
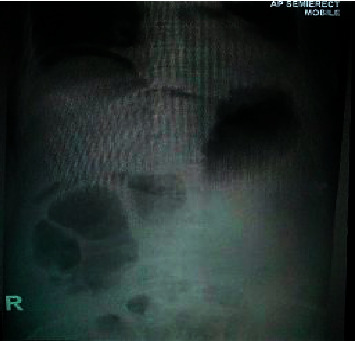
Plain X-ray abdomen; gas under diaphragm positive, gas-filled distended stomach, and some bowel loops.

On her 3rd week of treatment, diarrhea recurred and cardiac arrhythmias worsened. She was kept in an intensive care unit. Two days later, the patient suddenly developed severe pain in the right shoulder and right upper limb. In plain X-ray abdomen, gas was found under the diaphragm (Figure 6), and she was urgently referred to a surgeon for intestinal perforation. Unfortunately, her blood pressure and respiratory function were unstable, and we lost the patient before identifying the site of perforation in operation.

## 3. Discussion

The typical endoscopic findings in patients with UC include edematous mucosa, erythema, loss of vascular markings, and mucosal friability [11]. More severe cases may be associated with erosions, ulcers, and spontaneous bleeding. Luminal narrowing and pseudopolyps may occur due to chronic inflammation, which results in mucosal atrophy [12]. Although inflammation in UC patients is mostly limited to the colon, ileal inflammation (backwash ileitis) may infrequently be observed [13]. In this study, pseudopolyps present in both the upper GI tract and colon may be due to the chronicity of disease although the symptoms were there for 3 months only, and terminal ileum inflammation may be the backwash ileitis.

UC complicated with upper GI tract lesions is rare. An earlier study reported that two patients with UC developed duodenitis after total colectomy [14]. Subsequently, other studies have reported the same complication [7, 15–18]. The concept of GDUC was first proposed by Hori et al., but the diagnosis standard is not rigorous enough due to the scanty reports. More extensive colitis and a lower dose of prednisolone administration might be the main risk factors for GDUC [4]. Although the etiology of GDUC is unclear, several studies have revealed that it may be associated with the imbalance of immune response of genetically susceptible hosts to bacterial antigens, resulting in an excessive autoimmune response to the gastroduodenal epithelium [10]. The diagnostic criteria are lesions that do not respond to standard treatment for ulcers, such as H2 blockers and proton pump inhibitors, and lesions that are sensitive to medication used to treat UC, such as steroids and mesalazine [19].

Ulcerative gastroduodenal lesion (UGDL) and UC have similar histologic features, and previous reports have shown that many patients with UGDL, especially those with mild symptoms, respond positively to the treatment of UC with drugs such as sulfasalazine or mesalazine [9, 20]. For some patients with severe UGDL, either sulfasalazine or steroid hormones could not control the condition effectively, and the tumor necrosis factor (TNF) antagonist infliximab (IFX) or a calcineurin antagonist could be more effective in preventing the condition from deteriorating [20, 21]. To date, it is considered that early diagnosis with application of sulfasalazine or mesalazine is a quick and effective way to improve UGDL symptoms, and steroids could be used as a 2nd-line therapy for UGDL and the TNF antagonist IFX or a calcineurin antagonist for patients with rapid progress and hormone insensitivity. With the increasing number of upper GI mucosal lesions in patients with UC, we should pay attention to the pathogenesis and treatment of UGDL.

There are many evidences about GDUC which may be associated with progressing UC or total colitis, may have occurred in postcolectomy patients of UC, and may be associated with long-time lower dose of prednisolone. The present study suggests that GDUC was diagnosed since the diagnosis of UC. There were similar endoscopic findings and histological findings in both the upper GI tract and colon. This report may be the first case of synchronous UC of gastroduodenal and colonic lesion which is not reported yet.

The intestinal perforation represents the most serious complication in UC and is usually associated with instrumentation (colonoscopy) or with the presence of toxic megacolon and delay in diagnosis, due to the consequent presence of perforation [22]. In this study, perforation may represent the serious complication of UC and history of having taken low dose prednisolone for two months of duration. The patient did not start rescue therapy (IFX or cyclosporine) as she developed many clinical events such as cardiac arrhythmia, pancytopenia, and refeeding syndrome during periods, and bowel symptom was improved with mesalasine and steroid therapy.

## 4. Conclusions

UC may not be restricted to the large intestine; it is important to examine the upper gastrointestinal tract to confirm whether GDUC exists. Many case series and studies described that GDUC involvement in UC may be more frequent than previously estimated. The synchronous finding of UC in both gastroduodenal and colonic lesion may happen rarely. As for further studies involving a large number of adult patients, it needs to be clarified whether the upper gastrointestinal tract is a target organ in UC to better understanding the etiology and pathogenesis of UC.
